# MKRN1 degrades AGC1 to trigger chemotherapy resistance of colorectal Cancer

**DOI:** 10.1186/s10020-025-01287-2

**Published:** 2025-07-28

**Authors:** Yixuan Wang, Mu Qiao, Jing Guo, Ying Xie, Meilin Hu, Xin Li, Sheng Wang, Jingjing Wang, Jingya Wang, Ziyi Peng, Mengqi Wang, Hao Cheng, Tiantian Li, Linchuang Jia, Danchen Su, Huanhuan Liu, Kexin Hu, Xinyang Li, Wenjing Li, Di Wu, Zhe Zhang, Jianing Han, Ruiyang Bai, Funan Zhou, Zhiqiang Liu

**Affiliations:** 1 The province and ministry co-sponsored collaborative innovation center for medical epigenetics, Department of Physiology and Pathophysiology, School of Basic Medical Science, Tianjin Medical University, Heping, Tianjin, 300070 China; 2 Shandong Provincial Key Laboratory of Precision Oncology, Shandong Cancer Hospital and Institute, Shandong First Medical University, Shandong Academy of Medical Sciences, Jinan, 250117 Shandong China; 3 Tianjin Key Laboratory of Oral Soft and Hard Tissues Restoration and Regeneration, School of stomatology, Tianjin Medical University, Tianjin Medical University, Heping, Tianjin, 300070 China; 4 Department of Hematology, Tianjin Medical University Cancer Hospital, Tianjin Medical University, Heping, Tianjin, 300070 China; 5 Department of Geriatrics, The First Affiliated Hospital of Shandong Second Medical University , Weifang, 262400 Shandong PR China

**Keywords:** Colorectal cancer, Ubiquitination, MKRN1, AGC1, Heat shock proteins, Mitochondrial energy metabolism

## Abstract

Oxaliplatin (Oxa) has been extensively employed in treatment of colorectal cancer (CRC), yet frequent occurrence of chemoresistance poses a significant obstacle to achieving long-term disease-free survival for CRC patients. In this study, we employed a CRISPR/Cas9 sgRNA library targeting 1,117 human ubiquitination-related genes to screen key regulators of sensitivity to Oxa and identified the Makorin Ring Finger Protein 1 (MKRN1), an E3 ligase, as an Oxa-resistant gene of CRC cells. Clinically, MKRN1 is highly expressed in CRC tissues compared with the adjacent normal tissue, and its upregulation is correlated with poor therapeutic response, disease progression, and worse overall survival of CRC patients treated with Oxa-based regimens. In CRC cells, gain- and loss-of-function studies of MKRN1 respectively altered the sensitivity to Oxa treatment, as evidenced by changes in IC50 values and cell apoptosis. Mechanistic analysis revealed that MKRN1 interacts with Aspartate/Glutamate Carrier 1 (AGC1), facilitating degradation of AGC1 via K11- and K29-linked ubiquitination, thereby affecting mitochondrial function including energy metabolism and antioxidant responses. Through reprogramming of metabolic genes, this process enhances the expression of heat shock protein HSPD1 and HSP90AA1, while reducing oxidative stress, ultimately contributing to the development of Oxa resistance in CRC cells. Moreover, AGC1 knockdown rescued the MKRN1-deficiency induced Oxa-sensitivity of CRC cells and in xenograft mouse model. Translationally, we identified Rabdosiin as a potential inhibitor of MKRN1 through virtual screening, and validated the synergetic effect of Rabdosiin and Oxa in treating Oxa-resistant CRC cells both in vitro and in vivo. Taken together, our findings highlight the pivotal role of MKRN1-AGC1 axis in dictating CRC chemoresistance and offer novel therapeutic strategies for overcoming Oxa-resistance.

**Graphical Abstract:**

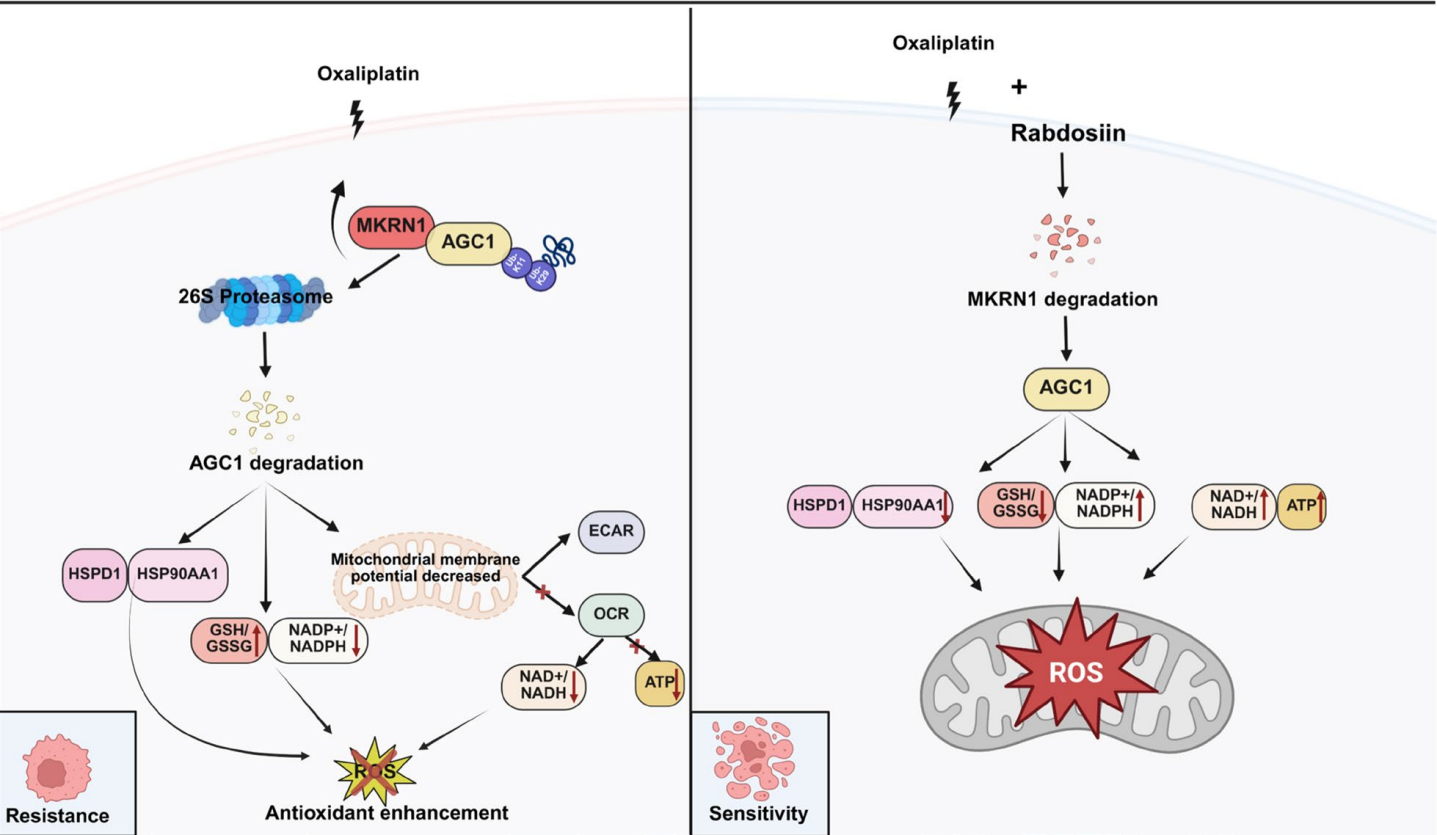

**Supplementary Information:**

The online version contains supplementary material available at 10.1186/s10020-025-01287-2.

## Introduction

Colorectal cancer (CRC) is one of the most common malignant tumors ranking as the fourth most frequently diagnosed cancer and the second leading cause of cancer death (Eng et al. 2024). The primary treatment for patients with colorectal cancer involves a combination of surgery and chemotherapy (Haynes and Manogaran 2025). In 2024, the NCCN (National Comprehensive Cancer Network) guidelines recommended the FOLFOX, CAPEOX and other regimens containing Oxaliplatin to treat CRC patients in the clinic (Benson et al. 2024). However, the emergence of drug resistance continues to pose a main obstacle in obtaining long-term progression-free survival for CRC patients. Hence, exploring molecular mechanisms underlying the chemoresistance is essential for eliminating this obstacle.

MKRN1, a member of the makorin family, contains conserved RING finger domains and is a well-defined E3 ubiquitin ligase (Huang et al. 2018). MKRN1 is primarily expressed in cytoplasm, regulates protein stability by catalyzing polyubiquitination. It has been reported to ubiquitinate and degrade key tumor suppressors, including p53/TP53 (Lee et al. 2009), PTEN (Lee et al. 2015), APC (Lee et al. 2018), and p14 ARF (Ko et al. 2012), for promoting tumorigenesis. High MKRN1 levels promote TGF-β signaling through ubiquitination and degradation of SNIP1, thereby facilitating CRC metastasis (Zhang et al. 2023). Moreover, evidence suggests that MKRN1 could be a potential target for treating metabolic diseases like obesity and diabetes (Kim et al. 2014). However, its precise roles in regulating CRC chemoresistance are not fully understood and require further investigation.

The malate-aspartate shuttle (MAS) facilitates the transport of glutamate from the cytoplasm into the mitochondria, while exporting mitochondrial aspartate and a proton into the cytoplasm via the aspartate-glutamate carrier (AGC). This process plays a crucial role in transferring electrons from cytosolic NADH to the mitochondria, with the exchange step being the only irreversible one in the MAS pathway (Thangaratnarajah et al. 2014). AGC1 (SLC25 A12, Aralar) is closely related to the occurrence and development of tumor cells. Deficiency of malate-aspartate shuttle component SLC25 A12 induces pulmonary metastasis (Alkan et al. 2020). However, the mechanism of AGC1 in tumor resistance remains unexplored.

In the current study, we aimed to identify key regulators for Oxa-resistance of CRC. We found MKRN1 was an Oxa-resistant gene, and high expression of MKRN1 was correlated with poor prognosis and treatment response. We further explored the mechanism of MKRN1 in regulating chemoresistance using in vitro and in vivo models, and finally we screened a specific inhibitor of MKRN1 and validated its combination effect with Oxaliplatin. Our findings suggest a potential therapeutic approach to overcome Oxa-resistance in CRC through targeting the MKRN1-AGC1 axis.

## Materials and methods

### Animals and treatment procedures

Animal studies were approved by the Committee on Animal Ethics and Welfare of Shandong First Medical University. All protocols conformed to the Guidelines for Ethical Conduct in the Care and Use of Nonhuman Animals in Research. For the in vivo experiments, MKRN1 or AGC1 deficient cells, or AGC1-overexpressing (OE) cells (2 × 10^6^) were subcutaneously injected into the abdominal region of 4–6-week-old nude mice (SPF Beijing Biotechnology, Beijing, China). Starting from day 7 post-cell injection, the mice were treated with oxaliplatin (Oxa), PBS, or Rabdosiin, twice a week, until humane endpoints were reached. Tumor volumes were measured every three days using calipers and calculated using the formula: [(length × width²)/2].

### Cell culture

Colorectal cancer cell line HCT116 was purchased from the National Infrastructure of Cell Line Resource (Beijing, China). Parental HCT116 cells and the Oxa-resistant cells (OR cells) were cultured in RPMI1640 media supplemented with 10% of fetal bovine serum (Gibco, Life Technologies, Carlsbad, CA, USA). Human embryonic kidney HEK293 T cells were plated with dulbecco’s modified Eagle’s medium (DMEM), including 10% fetal bovine serum (FBS), 100 U/ml penicillin, 100 µg/ml streptomycin. The medium was replaced every 2 days. All cells were STR authenticated (Biowing Biotech, Shanghai, China) and mycoplasma-free confirmed with the Universal Mycoplasma Detection Kit (ATCC, Manassas, VA, USA).

### Plasmid construction and viral infection

Specific oligonucleotides targeting human MKRN1 or AGC1, and non-target controls, were cloned into pLKO.1 vectors. For overexpression, MKRN1 or AGC1 cDNA was cloned into pITA-insert vectors. HEK293 T cells were transiently transfected using polyethyleneimine (PEI) (Polysciences, Warrington, USA) in OPTI-MEM medium (Life Technologies, Carlsbad, USA) with a ratio of 1:5 of DNA: PEI. Viral particles were produced by transfecting HEK293 T cells in 10 cm dishes with 4 µg PMD2G and 6 µg PSPAX2 packaging plasmids, together with 8 µg lentiviral expressing vectors encoding target genes (pITA-insert-hMKRN1-FLAG, MKRN1 knockdown, AGC1 knockdown, pITA-insert-hAGC1-FLAG). The viral supernatant was harvested and concentrated to 1/100 volume by PEG 8,000 (Sigma-Aldrich, St. Louis, USA). HCT116 cells (1 × 10^6^) were infected with concentrated lentivirus or vector controls using 200 µl viral concentrate and 8 µg/ml polybrene. Stable cells were selected with puromycin (SelleckChem, Houston, USA) after 72 h. Knockdown and overexpression efficiencies were confirmed by Western blots and qPCR.

### Screening of genes using CRISPR/cas9 SgRNA system

A CRISPR/Cas9 sgRNA library targeting 1,117 human ubiquitination related gene (Ub-library) was used to identify genes responsible for Oxa resistance and sensitivity in CRC cells. HCT116 cells were transfected with the Ub-library at a low multiplicity of infection (MOI ≤0.3). After selected with 1 µg/ml of puromycin, at least 8 million transduced cells were treated with 0, 2.5, 10 µg/ml Oxa for 72 h. After treatment, at least 3 replicate samples from each group were collected for genomic DNA extraction. Genomic DNAs of samples were extracted using the FastPure Cell/Tissue DNA Isolation Mini Kit (Vazyme, Nanjing, China). The sgRNA cassettes were amplified using NEBNext^®^ high-fidelity 2×PCR master mix and next generation sequencing was performed on an Illumina HiSeq to determine sgRNA abundance.

### RNA-sequencing and real-time RT-PCR assays

RNA sequencing was performed in the MKRN1- knockdown (KD) cells and AGC1-KD cells. Briefly, total RNA from treated samples was extracted using Trizol reagent (Life Technologies, Carlsbad, USA). The construction of the RNA-seq library and the sequencing were done by the Beijing Genomics Institute (Shenzhen, China). All mRNA samples were prepared for RNA-sequencing analysis according to the manufacturer’s protocol. For qPCR, total cellular RNA was extracted from cells with Trizol (Life Technologies, Carlsbad, USA) reagents and reverse transcription was performed. SYBR Green PCR kit (abm, Vancouver, Canada) was used to perform qPCR reactions. Each sample was repeated at least three times. *GAPDH* was used as the reference gene to normalize the expression of the target genes. For quantification of gene expression, the 2^−ΔΔCt^ method was used.

### Flow cytometry and cell viability assay

Annexin V-FITC (FITC-conjugated Annexin V) (eBioscience, San Diego, USA) was used to label apoptosis cells. Dead cells were labeled by PI (propidium iodide) (eBioscience, San Diego, USA). A straining experiment was performed according to the product instructions. Briefly, 1 × 10^6^ cells were washed in cold PBS and suspended in 0.5 ml staining binding buffer. Annexin V-FITC (5 µl) and PI (5 µl) were added to the cell suspension respectively. Cells were incubated for 15 min at room temperature and subjected to flow analysis. The results were analyzed using FlowJo software. For CCK8 assays, CRC cells were seeded at 1 × 10^5^ cells/well in triplicate in 96-well plates and were incubated at 37 °C with 5% CO_2_. After incubation for 48 h, CCK8 reagent was added to each well and incubated for 2 h prior to reading absorbance at 450 nm. The following formula was used to calculate cell viability (%) = OD value of treatment group/OD value of control group ×100.

### Immunofluorescence staining (IF)

Cells were fixed with 4% paraformaldehyde solution (Affymetrix, Santa Clara, USA) for 20 min, permeabilized with 0.1% (v/v) Triton X-100 for 10 min, and blocked with 5% BSA for 1 h. After incubation with primary antibody (1:100) overnight at 4 °C, washed with phosphate-buffered saline with tween 20 (PBST) 3 times for 5 min, and then incubated with a fluorescent secondary antibody coupled to Alexa-Fluor^®^ 488 goat anti-rabbit IgG (H + L) or Alexa-Fluor^®^ 594 goat anti-mouse IgG (H + L) for 60 min at room temperature and nuclei were counterstained with DAPI (Sigma-Aldrich, St. Louis, USA). Signal detection was carried out by fluorescence imaging performed using an Olympus FV1000 IX81-SIM Confocal Microscope (Olympus, Tokyo, Japan). All antibodies and vendors were provided in the key resources table.

### Western blotting and Co-Immunoprecipitation (Co-IP)

Cells were harvested in RIPA buffer with a protease inhibitor cocktail (APExBIO, Houston, USA), and histones were isolated by acid extraction. Briefly, cells were lysed on the ice and then total lysate (10 µg) was fractionated by SDS-PAGE in 4–12% polyacrylamide gels and transferred onto PVDF membrane. After blocking with 5% non-fat dry milk, membranes were incubated overnight at 4 °C with primary antibodies. Protein bands were visualized with HRP-conjugated secondary antibody for images captured by the ChemiDoc MP imaging system (Bio-rad, Hercules, USA). Co-IP was performed using the following antibodies and reagents: anti-MKRN1 (Bethyl Laboratories, Texas, USA, A300-990 A-M) and anti-FLAG M2 Affinity Gel (Sigma-Aldrich, St. Louis, USA, A2220), anti-AGC1 (Proteintech, Chicago, USA 26804-1-AP), rProtein A/G MagBeads (Yeasen, Shanghai, China, 36417ES08). IgG from Bethyl Laboratories was used as an endogenous Co-IP control. Briefly, MKRN1-OE HCT116 cells were seeded into a 10 cm dish at a density of 3 × 10^6^ cells. After 48 h, cells were lysed in native lysis buffer containing a PI cocktail for 30 min at 4 °C. Lysates were used for immunoprecipitation by 30 µl FLAG Gel antibody or incubated with 3 µg antibody and 30 µl rProtein A/G MagBeads of overnight, followed by incubation with Magbeads at 4 °C overnight. Immunoprecipitated proteins were used for immunoblotting with the indicated antibodies to identify the interacting proteins. All experiments were biologically replicated three times.

### Immunohistochemistry (IHC)

Tissue samples were formalin-fixed and paraffin-embedded. Slides were deparaffinized and rehydrated via successive immersion in the following solutions: 100% xylene I (10 min), 100% xylene II (10 min), 100% ethanol I (5 min), 100% ethanol II (5 min), 95% ethanol (5 min), 85% ethanol (5 min), 80% ethanol (5 min), 75% ethanol (5 min), 80% ethanol (5 min), double distilled water I (10 min), and double-distilled water II (10 min). 3% H_2_O_2_ solution was used to block deparaffinized tissue slides and a 10 mM citrate buffer (pH 6.0) was used to retrieve antigen. After successful blocking of the deparaffinized tissue, appropriately diluted primary antibodies were added onto the slides and incubated in a humidified chamber at 4 °C overnight, after which diluted biotinylated secondary antibodies were incubated for 1 h at room temperature. DAB substrate solution (Dako, Copenhagen, Denmark, K5361), which was newly made just before use, was utilized to reveal the color of antibody staining. Hematoxylin staining was used to localize Nuclei 1 to 2 min before mounting and capture.

### Tunnel assay

Tunnel assay was performed using the DeadEnd™ Fluorometric TUNEL System (Promaga, Tokyo, Japan). For paraffin-embedded sections, wash 3 times by 100% ethanol for 15 min at room temperature and then rehydrate samples by sequentially immersing the slides through graded ethanol washes (95%, 85%, 70% and 50%) for 3 min each at room temperature. Further wash the slides in PBS 3 times at room temperature and incubate slides with 100 µl of the 20 µg/ml proteinase K for 30 min at room temperature. Then they were washed and incubated with rTdT incubation buffer at 37 °C for 60 min in the dark. The samples were washed and stained by DAPI for 5 min at room temperature in the dark. Then the samples were washed three times and analyzed by the Olympus FV1000 IX81-SIM Confocal Microscope (Olympus, Tokyo, Japan).

### Mass spectrometry

Cells were resuspended in NP-40 lysis buffer (150 mM sodium chloride, 1% NP-40, 50 mM Tris PH 8.0) with protease inhibitor cocktail (Roche, Basel, Switzerland) and centrifuged at 12,000 g for 20 min at 4 °C. The supernatants were incubated with anti-FLAG M2 affinity gel at 4 °C overnight. After washing four times with NP-40 lysis buffer, flag protein complex was eluted with flag peptide (Sigma). The elites were resolved on NuPAGE 4–12% Bis-Tris gel (Invitrogen, Waltham, USA) and stained with a silver staining kit (Pierce, Waltham, USA). The protein bands were cut out and analyzed by liquid chromatography-tandem mass spectrometry (LC-MS/MS).

### Measurement of OCR and ECAR

Oxygen consumption rate (OCR) and extracellular acidification rate (ECAR) were determined using Seahorse XFp Analyzer (Agilent Technologies, Santa Clara, CA). One day prior to assay analysis, 10,000 cells were seeded in each well of a XF96e cell culture plate using culture media. Approximately 1 h before the assays, culture media was exchanged for 180 µl assay media. The plates were then incubated at 37 °C in a CO_2_-free incubator for 45 min–1 h prior to running the assay. OCR and ECAR were measured over 90 min (15 mix and measure cycles), with compounds being injected every 3 cycles. For the mitochondrial respiration assays, the following compounds were injected sequentially (final concentrations in the wells): oligomycin (3 µM), CCCP (0.5 µM), rotenone (1 µM), and antimycin A (AMA, 1 µM) (all compound reagents from Sigma-Aldrich, St. Louis, USA). For the glycolysis assays, the assay media was not supplemented with glucose and the following compounds were injected sequentially (final concentrations in the wells): glucose (10 mM), oligomycin (3 µM), and 2-deoxy-D-glucose (100 mM). Protein concentration was measured in each well for normalization using standard BCA assay (PanReac AppliChem, Darmstadt, Germany) according to manufacturer’s instructions (Kendzia et al. 2023).

### Measurement of metabolic state biomarkers

Mitochondrial membrane potential (C2006), ATP levels (S0027), GSH/GSSG ratio (S0053), NADP+/NADPH ratio (S0180S), and NAD+/NADH ratio (S0176S) were measured using kits from Beyotime (Jiangsu, China).

### Establishment of OR HCT116 cells

Establishment of Oxa-resistant CRC cells: Wild-type CRC cells were treated with 0.5 µg/ml Oxa, with the dose doubled monthly for five months to model the Oxa resistance process in clinical MM patients. Oxa resistance was monitored and confirmed using CCK-8 or flow cytometry. Cells with an IC50 value > 10 folds of the baseline were selected for further experiments.

### Statistical analysis

Statistical analysis was performed using the GraphPad Prism. The exact sample size (n) for each experimental group is provided in the figure legends and results section. Statistical significance was assessed using Student’s *t*-test. Tests were two-sided unless otherwise stated and the estimation of variation was determined. *P*-values are reported to indicate the significance of the results, with *P* < 0.05 considered statistically significant unless otherwise specified.

## Results

### CRISPR/cas9 SgRNA screening identifies MKRN1 as a potential drug-resistant gene

**Fig. 1:**
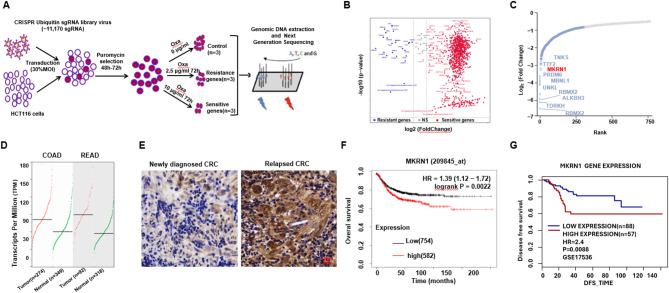
CRISPR/cas9 sgRNA screening identifies MKRN1 as a potential Oxa-resistant gene. **A** Screening strategy diagram for CRISPR/cas9 sgRNA library targeting ubiquitination-related genes in HCT116 cells. The successfully infected cells were positively selected using puromycin, and oxaliplatin (0 µg/ml, 2.5 µg/ml and 10 µg/ml) was employed to screen for drug-resistant and -sensitive genes (*n*=3). **B** Volcano plot illustrates the Oxa-resistant genes by negative selection and Oxa-sensitive genes by positive selection. **C** Illustration of the top 10 Oxa-resistant candidate genes. **D** The GEPIA2 online database illustrates the expression status of MKRN1 in colon and rectal cancer patients compared to normal individuals.**E** Immunohistochemical staining shows the protein expression of MKRN1 in newly diagnosed colorectal cancer patients (*n*=9) and relapsed colorectal cancer patients (*n*=9). Scale bar: 10μm. **F** Kaplan-Meier survival analysis for overall survival (OS) and (**G**) disease-free survival (DFS) for MKRN1 expression (high: red, low: blue) using data from the GenomicsScape online database. Survival distributions were compared via the Log-rank test. Cox regression models were employed to provide hazard ratios (HR) with 95% confidence intervals (CI), adjusting for variables such as age, gender, tumor stage, and treatment type.

We constructed a CRISPR/Cas9 sgRNA library targeting 1,117 human ubiquitination-related genes (UB-library) and assigned 6 distinct sgRNAs for each gene to minimize off-target effects. Following transduction with the pooled sgRNA library, HCT116 cells underwent puromycin selection. Subsequently, we treated the HCT116 cells with 2.5 µg/ml or 10 µg/ml Oxaliplatin (Oxa) (Fig. S1). The 2.5 µg/ml dosage showed no significant cytotoxic effect, whereas the 10 µg/ml dosage eradicated all wild-type tumor; thus, we can screen Oxa-resistant genes via negative selection in the 2.5 µg/ml-killed cells, while screen Oxa-sensitive genes via positive selection in the 10 µg/ml dosage-survived cells (Fig. 1A). We identified 887 Oxa-sensitive genes and 26 Oxa-resistant genes (Fig. 1B), in which *MKRN1* was observed in the Oxa-resistant gene. Further, the robust rank aggregation (RRA) algorithm confirmed *MKRN1* as one of the most essential genes (Fig. 1C). Data from GEPIA 2, which examines large TCGA and GTEx datasets, indicated that *MKRN1* was expressed at higher levels in colorectal cancers compared to normal tissues, respectively (Fig. 1D). Clinically, MKRN1 expression is elevated in post-treatment CRC patients compared to pre-treatment CRC patients (Fig. 1E). Colorectal cancer patients with higher *MKRN1* levels exhibited shorter overall survival and disease-free survival rates in the cohort GSE17536 (Fig. 1F and G). Collectively, these findings built a close correlation between *MKRN1* expression and chemoresistance of CRC.

### Gain- and loss-of-functions of MKRN1 alters chemoresistance of CRC cells

**Fig. 2:**
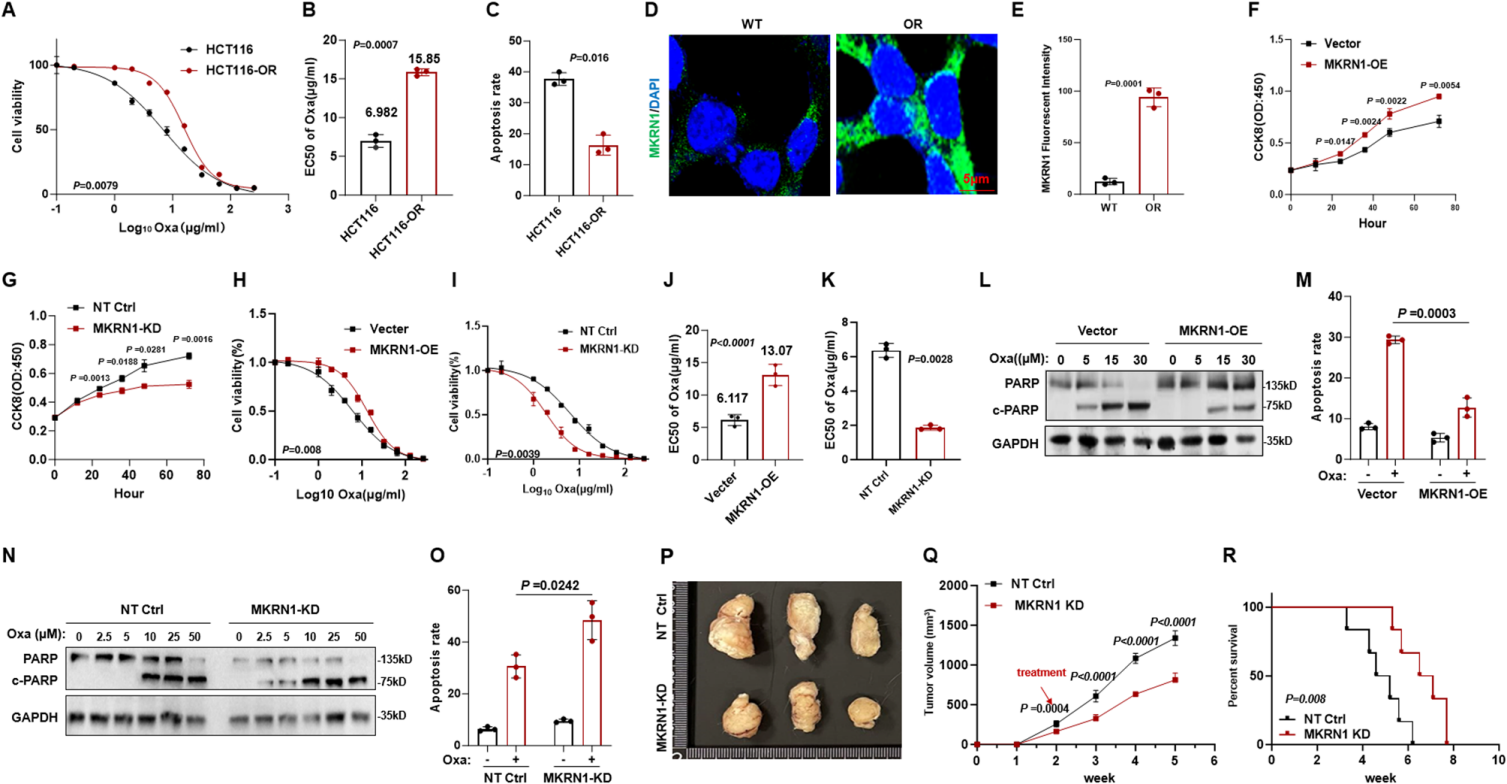
MKRN1 is upregulated in Oxa-resistant CRC cell. **A** The CCK8 assay was used to assess cell viability in wild-type (WT) and oxaliplatin-resistant (OR) HCT116 cells within 48 h (*n* = 3). **B** Changes in IC_50_ to oxaliplatin in WT and OR HCT116 cells. **C** The flow cytometry assay was conducted to detect apoptosis induced by 5 µM oxaliplatin for 48 h in WT and OR HCT116 cells. **D** Immunofluorescence staining of MKRN1 (green) and DAPI (blue) protein levels in OR HCT116 cells. Scale bar: 5 μm. **E** Quantification of MKRN1 protein intensity in WT and OR HCT116 cells. The CCK8 assay was used to assess cell proliferation (**F**,** G**) and cell viability (**H**,** I**) in MKRN1-OE and vector control, or MKRN1-KD and non-target control (NT Ctrl) HCT116 cells treated with oxaliplatin for 48 h, respectively (*n* = 3). **J**,** K** Changes in IC_50_ to oxaliplatin in MKRN1-OE and vector control, or MKRN1-KD and NT Ctrl HCT116 cells. **L** Western blotting assay shows the cleavage of PARP in MKRN1-OE and vector control HCT116 cells, and (**M**) shows apoptosis rates induced by 5 µg/ml oxaliplatin for 48 h. **N** Western blotting assay shows the cleavage of PARP in MKRN1-KD and NT Ctrl HCT116 cells, and (**O**) shows apoptosis rates induced by 5 µg/ml oxaliplatin for 48 h. **P** Tumor volume in 8-week-old nude mice subcutaneously injected with MKRN1 KD (*n* = 6) and NT Ctrl (*n* = 6) HCT116 cells (2 × 10^6^ cells/mouse) after approximately 4 weeks of treatment. Oxaliplatin (10 mg/kg) was administered approximately 1 week after injection and given every 2 days once tumors reached exceeded 15 mm. **Q** Tumor growth curve of MKRN1-KD (*n* = 6) and NC Ctrl (*n* = 6) HCT116 cells for 4 weeks post-treatment. **R** Survival rate of nude mice at the time points when tumor diameter exceeded 15 mm (*n* = 6). Scale bar: 5 μm. *P* values of mean ± SD were determined by Student’s *t* test (*n* = 6)

To further validate the role of MKRN1 in regulating chemosensitivity of CRC, we established the OR HCT116 cell lines in vitro. The OR cells displayed significant resistance to Oxa treatment, as evidenced by a markedly increased IC50 (Fig. 2A and B; Fig. S2 A) and considerably suppressed apoptosis (Fig. 2C). Additionally, the protein levels of MKRN1 were significantly elevated, as detected by immunofluorescence (Fig. 2D). Following the successful overexpression (OE) and knockdown (KD) of MKRN1 in HCT116 cell (Fig. S2 AC), it was observed that MKRN1-OE cells exhibited increased resistance and higher survival under Oxa treatment (Fig. 2E), while MKRN1-KD cells showed heightened sensitivity and reduced survival compared to the control groups, respectively (Fig. 2F). Additionally, MKRN1-OE cells demonstrated increased cell survival in response to Oxa treatment (Fig. 2G) and a significantly increased IC50 (Fig. 2H). This was accompanied by a marked reduction in cleaved PARP (Fig. 2I), a marker of apoptosis, and a notable inhibition of apoptotic cell rates (Fig. 2J). Conversely, cells with MKRN1-KD exhibited increased sensitivity to Oxa treatment (Fig. 2K), evidenced by a significantly decreased IC50 (Fig. 2L), increased PARP cleavage (Fig. 2M), and higher rates of apoptotic cells (Fig. 2N). In the MKRN1-KD and non-target control cells derived xenograft mice, we observed MKRN1-KD cell derived tumors exhibited a significantly repressed growth rate and smaller tumor size (Fig. 2O and P), as well as notably improved overall survival (Fig. 2Q). Taking together, these data indicate that MKRN1 is closely related with Oxa-resistance of CRC cells.

### MKRN1 regulates CRC chemosensitivity through interacting AGC1

**Fig. 3:**
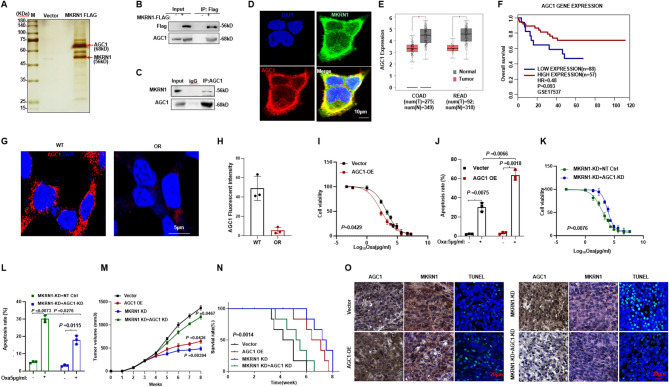
MKRN1 interacts with AGC1 in CRC cells. **A **Silver staining of flag-MKRN1 protein in HCT116 cells. **B-C** Exogenous and endogenous immunoprecipitation (Co-IP) assays demonstrate bilateral interactions between MKRN1 and AGC1. **D** Immunofluorescence analysis of AGC1 (red) and MKRN1 (green) in HCT116 cells, with nuclei stained by DAPI (blue). Scale bar: 10 μm. **E** The GEPIA2 online database shows the expression status of *AGC1* in colon and rectal cancer patients compared to normal individuals. **F** Kaplan-Meier survival curves for overall survival (OS) based on *AGC1* expression (high: red, low: blue) from the GenomicsScape online database. **G **Immunofluorescence of AGC1 (red) and DAPI (blue) to detect AGC1 expression in HCT116 cells. Scale bar: 5 μm. **H-I** The CCK8 assay was used to evaluate cell viability in AGC1-OE and vector, or **J-K** MKRN1 and AGC1 double knockdown HCT116 cells under increasing dosage of Oxa for 48 hours (*n*=3). Flow cytometry was performed to detect cell apoptosis induced by 5 μM Oxa for 48 hours in AGC1-OE and vector (**K**) or MKRN1 and AGC1 double knockdown HCT116 cells (*n*=3). **L** Tumor volume was measured in 8-week-old nude mice subcutaneously injected with AGC1 OE (*n*=6), vector (*n*=6), MKRN1-KD, and MKRN1-KD+AGC1-KD Oxa-resistant HCT116 cells (2×10^6^ cells/mouse) after approximately four weeks of treatment. Oxa (10 mg/kg) was administered approximately 1 week after injection and given every two days when tumors reached or exceeded 80 mm^3^ in size. **M** Tumor growth of HCT116 cells for 4 weeks post-treatment. **N** Survival rate of nude mice at the time points when tumor diameter exceeded 15 mm (*n*=6). **O** Immunofluorescence assay showing the levels of MKRN1 and AGC1 in tissues from xenografts of different mouse groups. Apoptosis was detected using a TUNEL kit. Scale bar: 50 μm. *P* values of mean ± SD were determined by Student’s *t* test *(n*= 6)

To elucidate the molecular mechanisms by which MKRN1 regulates CRC cell sensitivity to Oxa, we overexpressed a flag-tagged MKRN1 protein in HCT116 cells for immunoprecipitation assays to identify MKRN1-interacting proteins. Aspartate/Glutamate Carrier 1 (AGC1), a mitochondrial membrane-associated Ca^2+^-dependent aspartate-glutamate carrier (Palmieri et al. 2001), was identified as an interactor with MKRN1 through mass spectrometry analysis (Fig. 3A). Reciprocal exogenous (Fig. 3B) and endogenous (Fig. 3C) co-immunoprecipitation assays further confirmed the interaction, and immunofluorescence staining validated the co-localization of MKRN1 and AGC1 in the cytoplasm of CRC cells (Fig. 3D, Fig. S3 A). Clinically, *AGC1* expression was markedly decreased in colon and rectal cancer tissues compared to normal tissues in the GEPIA2 online database (Fig. 3E), and patients with lower *AGC1* expression exhibited significantly shorter overall survival time (Fig. 3F). As expected, AGC1 protein was also suppressed in the OR-HCT116 cells (Fig. 3G, Fig. S3B). When overexpressing AGC1 in HCT116 cells (Fig. S3 C), AGC1-OE cells demonstrated a substantial decrease in cell viability (Fig. 3H) and a significant increase in cell apoptosis when exposed to Oxa (Fig. 3I). In contrast, subsequential knockdown of AGC1 in the MKRN1-KD cells (Fig. S3D) rescued the sensitivity of MKRN1-KD cells to Oxa (Fig. 3J) and reduced the rate of apoptotic cells (Fig. 3K). To validate the rescue effect of AGC1 on sensitivity to Oxa of the MKRN1-KD cells in vivo, we injected the AGC1-OE, MKRN1-KD, or MKRN1-KD with AGC1-KD HCT116 cells into nude mice and administered the mice with 10 mg/kg of Oxa every 2 days. Compared with tumors derived from the above four groups, AGC1-OE or MKRN1-KD plus AGC1-KD CRC cells showed significantly increased tumor growth, prolonged overall survival rate, and decreased apoptosis rates (Fig. 3M and O). These in vivo data, along with the above in vitro results, indicate that MKRN1 expression impacts chemosensitivity of CRC cells.

### MKRN1 catalyzes AGC1 polyubiquitination at K11 and K29

**Fig. 4:**
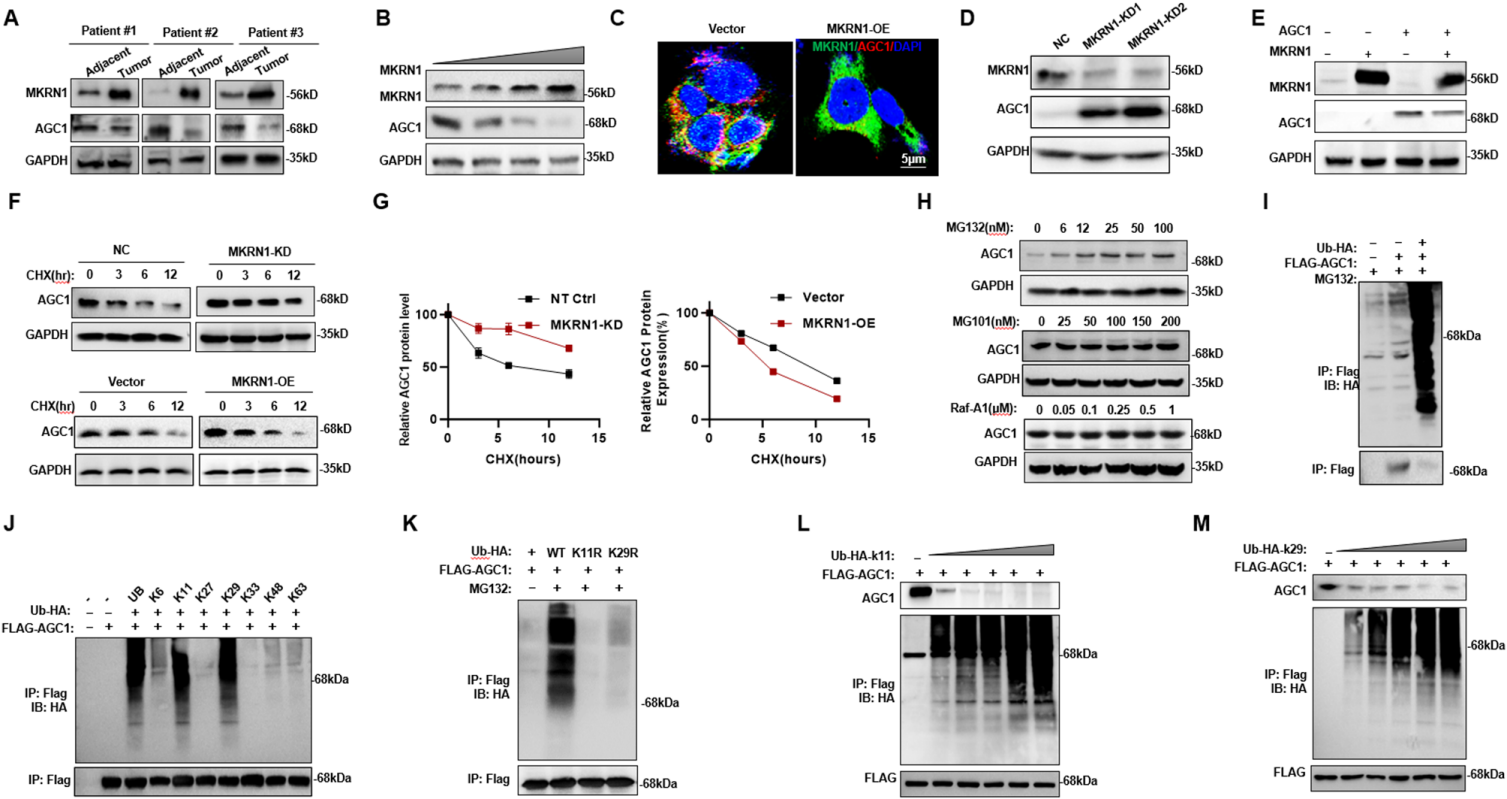
MKRN1 degrades AGC1 via K11- and K29-linked polyubiquitination. **A** Protein expression levels of MKRN1 and AGC1 proteins in CRC tissues and adjacent normal tissues of relapsed patients. **B** AGC1 protein expression in HCT116 cells with gradient overexpression of MKRN1. **C** Fluorescence colocalization of AGC1 (red) and MKRN1 (green) in HCT116 cells with vector control and MKRN1 overexpression. **D** AGC1 protein expression in HCT116 cells with MKRN1 knockdown. **E** AGC1 and MKRN1 protein expression in HCT116 cells with simultaneous overexpression of both proteins. **F-G** Evaluation of AGC1 protein degradation rate in vector control and MKRN1 overexpression, or NC and MKRN1 knockdown HCT116 cells treated with 20 µM cycloheximide (CHX) for varying durations. **H** AGC1 protein expression in HCT116 cells treated with gradient concentrations of proteasome inhibitor MG132, lysosomal inhibitor MG101, and autophagy inhibitor baf-A1. **I** Co-immunoprecipitation assays demonstrating the ubiquitination of AGC1 in HCT116 cells transfected with flag-AGC1 and HA-ubiquitin, with or without 10 µM MG132 for 6 h. **J** Investigation of key lysine residues involved in the polyubiquitination of AGC1 proteins in HCT116 cells transfected with flag-AGC1 or various positively mutated HA-ubiquitin constructs, in the presence of 10 µM MG132 for 6 h. **K** Analysis of ubiquitination of flag-AGC1 in HCT116 cells transfected with negatively mutated HA-ubiquitin constructs in the presence of 10 µM MG132 for 6 h. **L-M** Evaluation of flag-AGC1 levels in HCT116 cells co-transfected with gradient concentrations of Ub-K11 or Ub-K48 for 48 h

As an E3 ligase, MKRN1 is supposed to catalyze and degrade the substrate AGC1 through ubiquitination. Indeed, we observed in rectal cancer tissues from patients who did not response to Oxa-based chemotherapies, MKRN1 expression was elevated while AGC1 was repressed (Fig. 4A). Importantly, overexpressing MKRN1 in HCT116 cells led to the degradation of AGC1 protein at a dose-dependent manner (Fig. 4B), accompanying disappear of colocalization in cytoplasm (Fig. 4C). Conversely, knocking down MKRN1 in HCT116 cells resulted in increased expression of AGC1 protein (Fig. 4D). Overexpression of AGC1 prevented the degradation of AGC1 by MKRN1 (Fig. 4E). By treating cells with cycloheximide (CHX) to inhibit protein synthesis and monitoring changes in protein levels, we observed that MKRN1-KD enhanced the stability of AGC1, while MKRN1-OE increased the degradation rate of AGC1, thereby reducing its stability (Fig. 4F and G). When analyzing major pathways for protein degradation, we found only the proteasome inhibitor MG132, but not the protease inhibitor MG101 or the lysosomal inhibitor Baf-A1, resulted in obvious accumulation of AGC1 protein, indicating that AGC1 degradation was mainly proteasome-dependent (Fig. 4H). Through co-expression of HA-tagged ubiquitin and flag-tagged AGC1, we observed that AGC1 protein could be modified by polyubiquitination (Fig. 4I). Using a series of positive mutations on lysine residues of ubiquitin, we identified that polyubiquitination of AGC1 protein was mainly through K11- and K29-linked ubiquitin, which are key mediators for degradation (Fig. 4J). Conversely, when K11 and K29 were negatively mutated, ubiquitination of AGC1 protein was barely detected (Fig. 4K). Overexpression of ubiquitin with K11- and K29- positive mutations enhanced the polyubiquitination and degradation of AGC1 in a dose-dependent manner (Fig. 4L and M). Thus, these results strongly suggest that E3 ligase MKRN1 catalyzes AGC1 polyubiquitination for degradation.

### AGC1 triggers oxidative stress in mitochondria of CRC cells

**Fig. 5:**
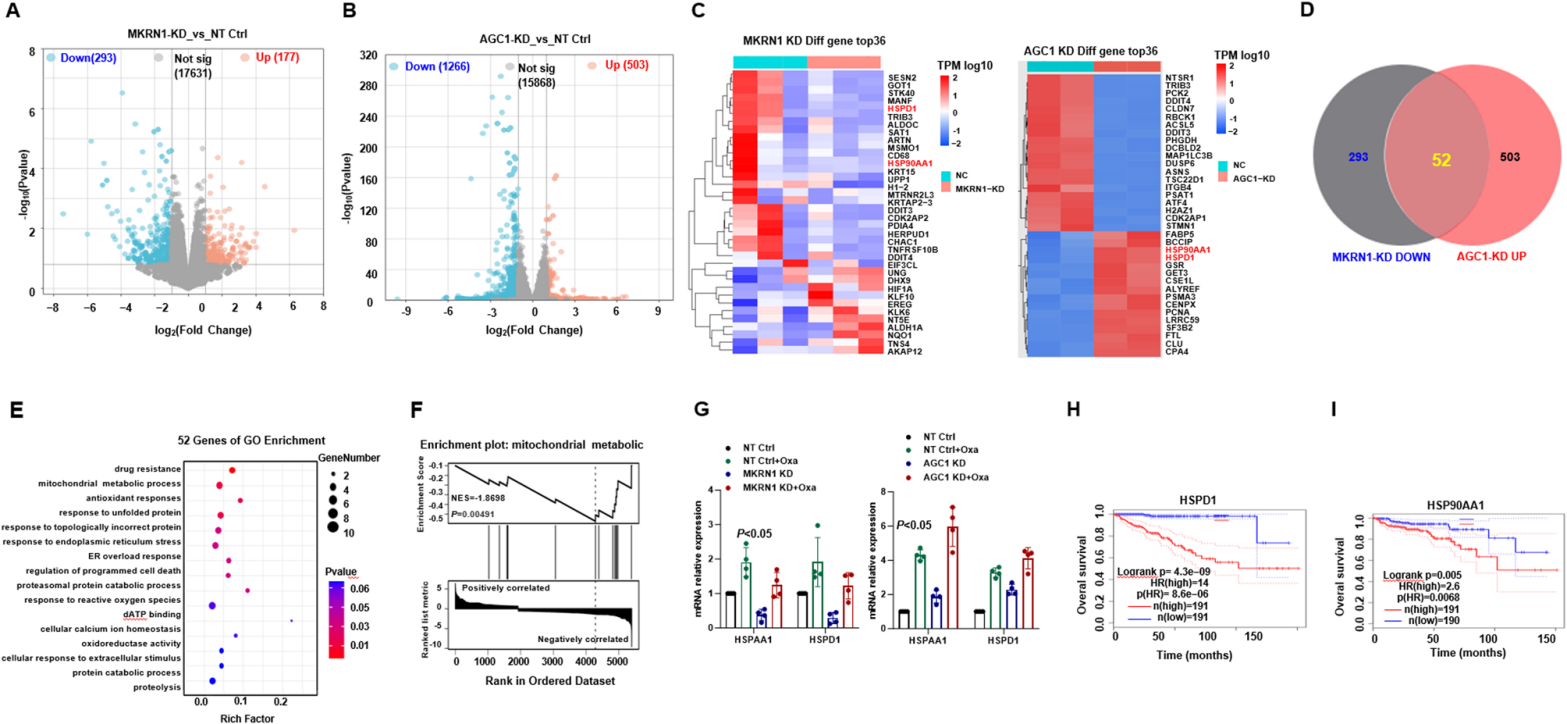
Transcriptomic analysis reveals changes in antioxidant and mitochondrial metabolism genes following MKRN1 and AGC1 knockdown. **A-B **Volcano plots of differentially expressed genes from bulk RNA-sequencing in MKRN1-KD and AGC1-KD HCT116 cells. Blue dots represent downregulated genes, red dots represent upregulated genes, and gray dots represent statistically non-significant genes. **C** Heatmaps displaying the expression levels of the top 36 differentially expressed genes in MKRN1-KD and AGC1-KD groups. **D** Venn diagram showing the overlap between differentially expressed genes in the MKRN1-KD downregulated (293 genes) and AGC1-KD upregulated (503 genes) groups. **E** Gene Ontology (GO) enrichment analysis for the 53 differentially expressed genes with *P* < 0.05, using DAVID methods. **F** Gene Set Enrichment Analysis (GSEA) assessing the enrichment of mitochondrial metabolic functions in the experimental groups, NES = -1.8698, *P* < 0.05. **G** HSPD1 and HSP90AA1 mRNA expressions in NT Ctrl, MKRN1-KD, and NT Ctrl, AGC1-KD HCT116 cells. **H** Kaplan-Meier survival curves for colorectal cancer patients based on HSPD1 and HSP90AA1 expression levels, using the GEPIA2 online database. *P* values of mean ± SD were determined by Student’s *t* test (*n*=3)

Oxaliplatin generates a large amount of reactive oxygen species (ROS) through redox reactions, which exacerbates DNA damage and induces cell apoptosis (Lin et al. 2022). Given that AGC1 lies downstream of MKRN1 regulation, we performed RNA-sequencing for both MKRN1-KD and AGC1-KD HCT116 cells (Table S1−4), and through crosslinking the differentially expressed genes (DEGs), we identified downstream targets co-regulated by both factors. The MKRN1-KD yielded 293 downregulated genes and 177 upregulated genes, while AGC1-KD produced 1266 downregulated genes and 503 upregulated genes (Fig. 5A and B). Analysis of the top DEGs in both MKRN1-KD and AGC1-KD cells, a significant accumulation of heat shock proteins was observed (Fig. 5C). We further enriched 293 downregulated genes from MKRN1-KD group and 503 upregulated genes from AGC1-KD group, identifying 52 common genes between the two groups (Fig. 5D). Gene Ontology (GO) enrichment analysis of these 52 genes highlighted significant involvement in mitochondrial metabolism, drug resistance, and antioxidant responses (Fig. 5E). Gene Set Enrichment Analysis (GSEA) also revealed a marked downregulation of mitochondrial metabolic functions in the experimental group (Fig. 5F). Among these genes, *HSPD1* and *HSP90 AA1* were selected for further investigation due to their prominent differential expression. Both *HSPD1* and *HSP90 AA1* mRNA levels were significantly upregulated in MKRN1-KD and AGC1-KD CRC cells upon Oxa treatment (Fig. 5G). Clinically, data from the GEPIA 2 database indicated that higher expression levels of *HSPD1* and *HSP90 AA1* are associated with shorter overall survival in colorectal cancer patients (Fig. 5H and I).

### MKRN1-AGC1 axis shift metabolic reprogramming of CRC cells towards Glycolysis

**Fig. 6:**
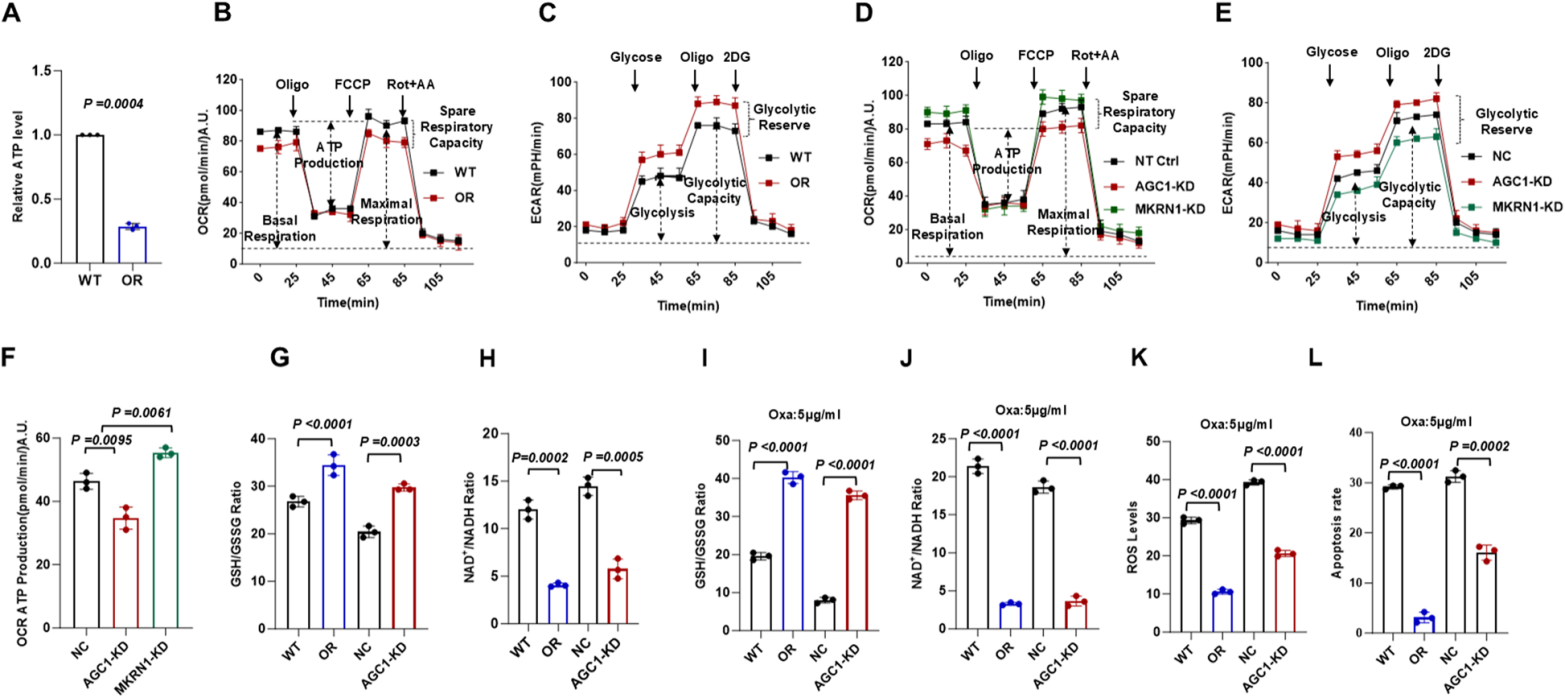
MKRN1-AGC1 axis shift metabolic reprogramming of CRC cells towards glycolysis. **A** Relative ATP amount was measured in the wild type (WT) and Oxa-resistant (OR) HCT116 cells. **B** Oxygen consumption rate (OCR) and (**C**) extracellular acidification rate (ECAR) were measured using a Seahorse XF24 analyzer in wild type (WT) and Oxa-resistant (OR) HCT116 cells. **D** OCR and (**E**) ECAR were measured using a Seahorse XF24 analyzer in non-target control (NC) and MKRN1-KD or AGC1-KD HCT116 cells. **F** Quantification of ATP amount measured in was measured in the OCR of non-target control (NC) and MKRN1-KD or AGC1-KD HCT116 cells. **G** GSH/GSSG ratio and **H **NADP^+^/NADPH ratio were measured in AGC1-KD and NT control or WT and OR HCT116 cells. **I** GSH/GSSG ratio and (**J**) NADP^+^/NADPH ratio were measured in AGC1-KD and NT control or WT and OR HCT116 cells treated with 5 µg/ml Oxa for 48 h. **K** ROS production was measured in AGC1-KD and NT control or WT and OR HCT116 cells treated with 5 µg/ml Oxa for 48 h. **L** Apoptosis rate of AGC1-KD and NT control or WT and OR HCT116 cells treated with 5 µg/ml Oxa for 48 h. *P* values of mean ± SD were determined by Student’s t test (*n* = 3)

In the Oxa-resistant CRC cells, we observed metabolic behaviors that diverge fundamentally from canonical energy metabolism, that the Oxa-resistant cells demonstrated a significant reduction in ATP synthesis efficiency (Fig. 6A). To further elucidate the impact of Oxa-resistance on mitochondrial oxidative phosphorylation and glycolytic metabolism, we quantified both the extracellular acidification rate (ECAR) and oxygen consumption rate (OCR). Indeed, we found Oxa-resistant cells displayed markedly lower basal respiration, ATP production, maximal respiratory capacity, and spare respiratory capacity compared to the WT cells (Fig. 6B). In contrast, Oxa-resistant cells exhibited significantly elevated basal glycolytic rate and glycolytic capacity, suggesting that these cells underwent metabolic reprogramming and shifted towards glycolysis, a hallmark of the Warburg effect (Fig. 6C). We then analyzed MKRN1-KD and AGC1-KD CRC cells, which respectively correspond to oxaliplatin-sensitive and oxaliplatin-resistant phenotypes. Our results demonstrated that AGC1-KD cells phenocopied the Oxa-OR characteristics, whereas MKRN1-KD CRC cells exhibited opposing metabolic features (Fig. 6D-F). We subsequently focused on metabolic shift exhibited by AGC1-KD CRC cells, which mimicking the Oxa-resistance, at the baseline or under Oxaliplatin treatment stress, through monitoring the ratio of GSH to GSSG and the NADP+/NADPH ratio. AGC1-KD cells and OR cells demonstrated markedly higher GSH/GSSG ratio (Fig. 6G) and decreased NADP+/NADPH ratio (Fig. 6H), compared with their controls (*P* < 0.05, *P* < 0.005, respectively). Under short-term Oxa treatment, both AGC1-KD and Oxa-resistant CRC cells still exhibited a stronger antioxidant stress capacity, as evidenced by a higher GSH/GSSG ratio (Fig. 6I), a lower NAD+/NADH ratio (Fig. 6J), a less ROS accumulation (Fig. 6K), and stronger anti-apoptosis capacity (Fig. 6I), suggesting that AGC1-KD cells and Oxa-resistant cells exhibit enhanced antioxidant capacity. Overall, these data collectively suggest that MKRN1-AGC1 axis dictates metabolic reprogramming in CRC cells, promoting a glycolytic shift under oxaliplatin pressure.

### Targeting MKRN1 re-sensitizes Oxa-sensitivity of CRC cells

**Fig. 7:**
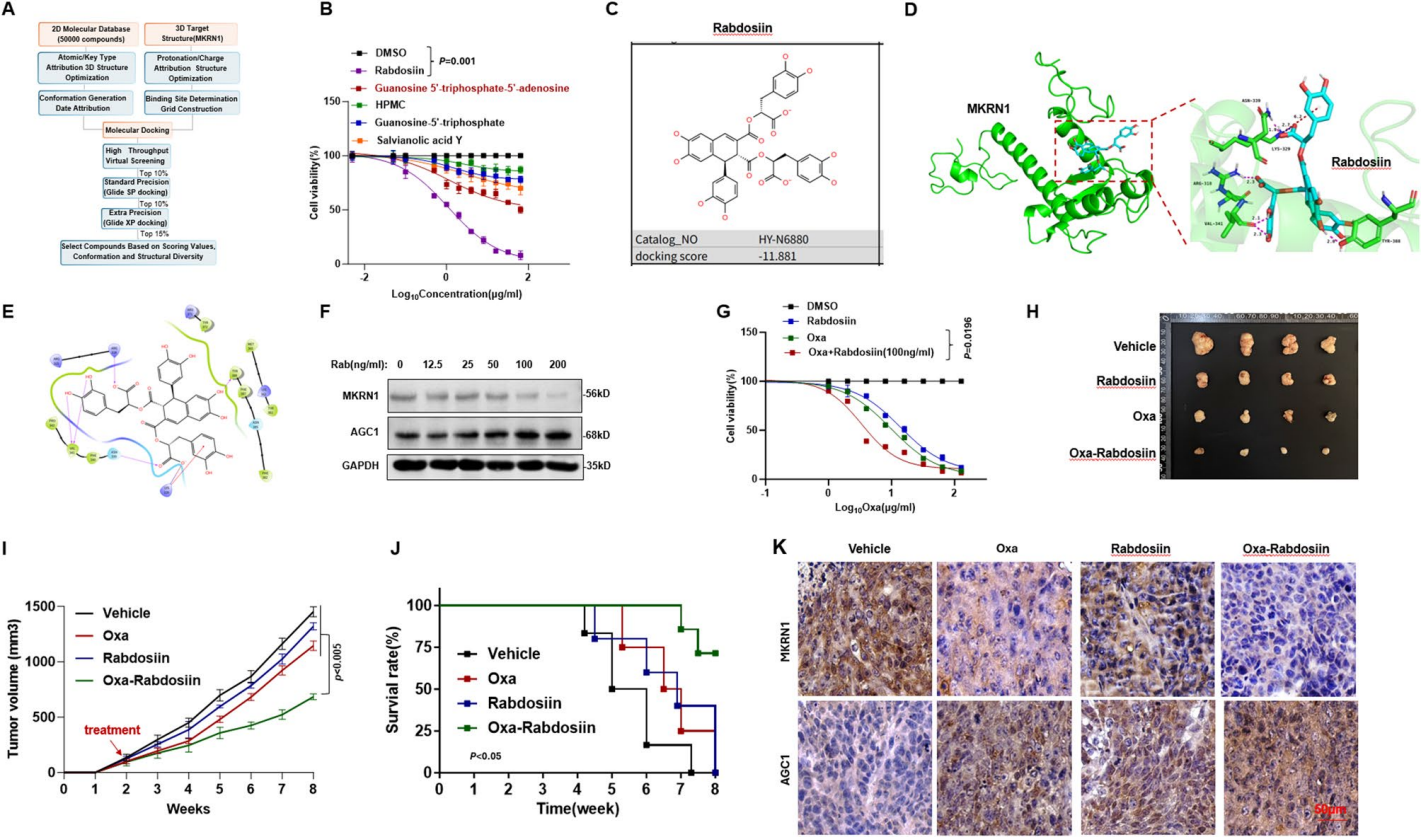
Identification of MKRN1 specific inhibitor and evaluation of the effect on overcoming Oxa-resistance in vivo. **A** Schematic flow chart of virtual screening MKRN1 inhibitor. **B** CCK-8 assay was used to evaluate cell viability in HCT116 cells treated with the top 5 compounds from virtual screening for 48-hour period (*n* = 3). **C** Chemical structure of Rabdosiin. **D-E** 3D and 2D binding patterns of Rabdosiin and MKRN1 protein. **F** MKRN1 and AGC1 protein levels in HCT116 cells treated with gradient concentrations of Rabdosiin for 48 h. **G** CCK-8 assay was used to evaluate cell viability in HCT116 cells treated with 5 µg/ml Oxaliplatin and gradient concentrations of Rabdosiin for 48 h (*n* = 3). **H** Tumor volume in 8-week-old nude mice subcutaneously injected with HCT116 cells (2 × 10^6^ cells/mouse) after approximately four weeks of treatment. Oxaliplatin (10 mg/kg) and Rabdosiin (1 mg/kg) were administered approximately one week after injection and given every two days when tumors reached or exceeded 80 mm^3^ in size. **I** Tumor growth rates following treatment with either Oxaliplatin alone, Rabdosiin alone, or their combination. **J** Survival rate of nude mice at the time points when tumor diameter exceeded 15 mm (*n* = 6). **K** Immunofluorescence assay showing the levels of MKRN1 and AGC1 in tissues from xenografts of different mouse groups treated with either Oxaliplatin alone, Rabdosiin alone, or their combination. Scale Bar: 50 μm. *P* values of mean ± SD were determined by Student’s t test (*n* = 6)

Although functions of MKRN1 in regulating CRC chemoresistance has been established, there is still lacking small molecule compounds specifically targeting MKRN1. To address this gap, we conducted virtual screening using the 3D structure of human MKRN1, with allosteric sites predicted by AlphaFold (AF-Q9UHC7-F1). Notably, we focused on Site 1 of MKRN1, which was predicted by Schrödinger software and involves key amino acids including VAL341, LYS362, ASN339, and CYS386. We then performed virtual docking of over 50,000 compounds into this predicted binding site (Fig. 7A). Based on the top-ranked MKRN1-compound binding models, we selected five promising compounds after a comprehensive evaluation of docking scores, lipophilicity, and water solubility. Among these, Rabdosiin was chosen for further study (Fig. 7B-C). The 2D and 3D docking patterns revealed that the hydroxyl groups of Rabdosiin formed three hydrogen bonds with VAL341 and TYR388 sites of MKRN1 protein. Additionally, the carboxyl group of Rabdosiin formed two hydrogen bonds with ASN339 and ARG318 of MKRN1, as well as one salt bridge with LYS329 and one π-cation interaction (Fig. 7D-E). Furthermore, increasing dosage of Rabdosiin treatment resulted in augmentation of AGC1 protein at a dose-dependent manner (Fig. 7F). In CRC cells, Rabdosiin in combination with Oxa further meaningfully inhibited cell viability (Fig. 7G).

To assess the therapeutic potential in vivo, we established Oxa-resistant CRC cells derived xenograft model for receiving administration of vehicle, Oxa, Rab, or combination of Oxa and Rab. Results showed that, compared to the solo administration groups, tumors from the combined treatment group exhibited obvious smaller volumes (Fig. 7F), significantly reduced tumor growth (*P* < 0.05) (Fig. 7G), and markedly improved survival rates (*P* < 0.05) (Fig. 7H). Immunohistochemical analysis of the tumors revealed a significant increase in AGC1 expression and a marked decrease in MKRN1 and HSP90 AA1 levels in tumors from the combination treatment group (Fig. 7I). These data collectively indicate that tarting MKRN1 is a promising approach to overcome Oxa-resistance of CRC.

## Discussion

Chemoresistance represents a significant clinical challenge that severely affects clinical outcomes of CRC patients, thus decoding the molecular mechanisms underlying Oxa-resistance may pave a way for improving chemotherapy efficacy (Luo et al. 2024). In this study, we employed a CRISPR/Cas9 sgRNA library targeting 1,117 human ubiquitination-related genes to screen key regulators of sensitivity to Oxa. We identified MKRN1 as an Oxa-resistance gene and elucidated its role as an E3 ubiquitin ligase that affects mitochondrial function, including energy metabolism and antioxidant responses in CRC. Mechanistically, MKRN1 facilitates the degradation of AGC1 via K11- and K29-linked ubiquitination, subsequently enhances the expression of heat shock proteins HSPD1 and HSP90 AA1 while reducing oxidative stress. Translationally, we identified Rabdosiin as a potential MKRN1-targeting compound, offering a promising strategy to overcome chemotherapy resistance in CRC. Thus, our findings provide new insights into the origins of chemoresistance in CRC patients and suggest novel approaches for developing therapies aiming at reducing CRC resistance.

In the current investigation, MKRN1 was found to be highly expressed in the recurrent CRC patients, and elevated MKRN1 expression promoted Oxa-resistance. MKRN1, a member of the RNF family of proteins, has a N-terminal RING structural domain that interacts with ubiquitin and transports it to the protein substrate, like other RING finger proteins (Carpenedo et al. 2016). Previous studies have revealed that MKRN1 plays critical roles in progression of various cancers. For example, SF3 A2 promotes progression and cisplatin resistance in triple-negative breast cancer via alternative splicing of MKRN1 (Deng et al. 2024); MKRN1 promotes colorectal cancer metastasis by activating the TGF-β signaling pathway through SNIP1 protein degradation (Zhang et al. 2025); CircVPS8 promotes malignant phenotypes and inhibits ferroptosis of glioma stem cells by acting as a scaffold for MKRN1 (Hu et al. 2024). In the current investigation, we discovered that MKRN1 acted as a resistance gene in CRC cells using optimized sgRNA library, as its depletion sensitized CRC cells to Oxa. Further in vitro and in vivo validations revealed that high MKRN1 expression was closely correlated with Oxa-resistance of CRC cells, and clinical data also confirmed that MKRN1 expression levels negatively correlated with patient response to Oxa treatment. Thus, our study identifies MKRN1 as an important biomarker for CRC chemoresistance.

The ubiquitin-proteasome system (UPS) is well-known to play a critical role in tumor chemoresistance, primarily by regulating the stability of key proteins, protein-protein interactions, and the subcellular localization of proteins (Dewson et al. 2023). MKRN1, an E3 ubiquitin ligase, is implicated in cancer biology by binding to and triggering the degradation of target proteins (Hoeller et al. 2006). Our study uncovered that AGC1 is a novel substrate for MKRN1 ubiquitination, and we identified that AGC1 was polyubiquitinated through K11- and K29-linked ubiquitination, altering its protein stability, with the key ubiquitination residues responsible for AGC1 degradation. AGC1 is a mitochondrial solute carrier protein of the SLC25 family, and has been reported to play pivotal role in the malate-aspartate shuttle by exchanging aspartate from the mitochondrial matrix with glutamate and protons from the cytoplasm, driven by a bioenergetic PH gradient across the inner mitochondrial membrane (Rabionet et al. 2006). As the primary mitochondrial aspartate exporter, AGC1 regulates redox potential between the mitochondria and the cytoplasm (Kanellopoulos et al. 2020). Moreover, our clinical analysis further confirmed that AGC1 expression was positively correlated with patient response to Oxa-based chemotherapies, with higher AGC1 expression associated with better survival outcomes. By crosslinking the transcriptomes of the MKRN1- and AGC1-deficient CRC cells, we identified that DEGs were primarily enriched in pathways related to mitochondrial dysfunction, antioxidant responses, and tumor drug resistance. Since Oxa primarily induces cell growth arrest and apoptosis through ROS-mediated damage, these findings suggest that targeting the MKRN1-AGC1 axis may enhance CRC cell sensitivity to Oxa via ROS accumulation.

Our findings deviate from conventional mitochondrial energy metabolism, as we observed reduced ATP synthesis in Oxa-resistant CRC cells. This may be due to the alteration of the cellular metabolic balance by AGC1 deficiency, which enables the cells to compensate for energy deficits by enhancing glycolysis, thus reducing their reliance on oxidative phosphorylation. Additionally, the increased GSH/GSSG ratio and decreased NADP^+^/NADPH ratio indicated enhanced antioxidant capacity. We hypothesize that this may result from metabolic reprogramming, wherein CRC cells actively suppress mitochondrial function and downregulate oxidative phosphorylation, sacrificing ATP production efficiency for survival advantage. Mechanistically, AGC1 participates in mitochondrial aerobic respiration by shuttling NADH into mitochondria for the oxidative respiratory chain. However, in the Oxa-resistant CRC cells, elevated MKRN1 expression leads to degradation of its target protein AGC1, consequently reducing AGC1 protein levels and oxidative phosphorylation activity. Moreover, the activation of the MKRN1-AGC1 axis promoted the upregulation of HSPD1 and HSPAA1(Wang et al. 2023), further inhibiting ROS generation. By cross-linking the transcriptomes of MKRN1- and AGC1-deficient CRC cells, a significant accumulation of heat shock proteins was observed. Specifically, activation of the MKRN1-AGC1 axis promotes the upregulation of HSPD1 and HSP90 AA1, consequently promotes ROS generation, thereby triggering chemosensitivity of CRC cells. Ultimately, these metabolic reprogramming changes contributed to CRC cell resistance to Oxa and drove adaptive metabolic remodeling.

The translational significance of this study lies in our identification and validation of a specific MKRN1 inhibitor. Through virtual screening, we identified Rabdosiin (Ito et al. 1998) as an MKRN1 inhibitor that enhances CRC cell sensitivity to Oxa. Experimental results demonstrated that Rabdosiin treatment could upregulate AGC1 expression, thereby sensitizes CRC cells to Oxa in our in vitro and in vivo models. Thus, targeting the MKRN1-AGC1 axis could reverse this process, enhancing cytotoxicity of Oxa and improving chemotherapy efficacy in CRC patients. This discovery sheds light on developing promising clinical targets for novel CRC therapeutic strategies.

In conclusion, we propose in Oxa-resistant colorectal cancer, elevated MKRN1 promotes AGC1 degradation via K11- and K29-linked ubiquitination, leading to proteasomal degradation, enhancing heat shock protein expression, and reducing oxidative stress, thereby conferring Oxa resistance. Moreover, we identified Rabdosiin as a promising MKRN1-targeting compound, which offers a potential strategy to overcome chemotherapy resistance in CRC.

## Supplementary Information

Supplementary Material 1.

## Data Availability

No datasets were generated or analysed during the current study.
