# Supplementary material for: MKRN1 degrades AGC1 to trigger chemotherapy resistance of colorectal Cancer
Source: Mol Med. 2025 Jul 28;31:268. doi: 10.1186/s10020-025-01287-2 (PMC12302804; doi:10.1186/s10020-025-01287-2)

**Supplement figure 1**

**Fig. S1 Detection of the sensitivity of HCT116 cells to oxaliplatin.**

**(A)** HCT116 cells were treated with gradient concentrations of oxaliplatin for 48 hours, and cell viability was assessed using the CCK-8 assay. It was observed that cell death began at a concentration of 2.5 μg/ml, with complete cell death occurring at a minimum lethal concentration of 10 μg/ml. The EC50 was determined at 4.327 μg/ml.


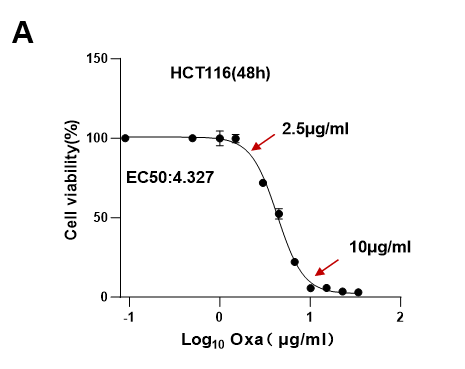


**Fig. S2 The efficiency of MKRN1 and AGC1 overexpression or knockdown.**

Western blotting assay was used to detect: **(A)**the expression of MKRN1 in the wild type (WT) or Oxa-resistant (OR) HCT116 cells; **(B)** MKRN1 expression in HCT116 cells overexpressing vector control or Flag-MKRN1 fusion protein; **(C)** Efficiency of MKRN1 knockdown using lentiviral carrying shRNA.


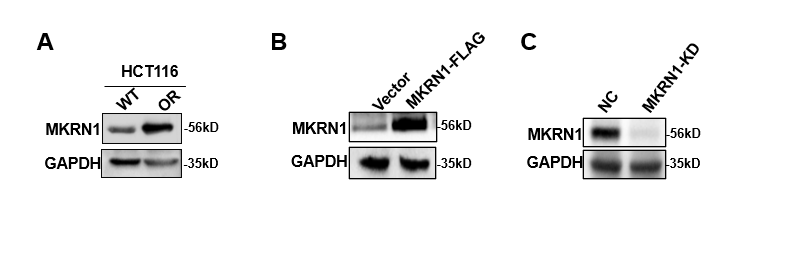


**Fig. S3 Co-localization of AGC1 and MKRN1 with mitochondria and efficiency of AGC1 expression.**

**(A)** Immunofluorescence assay for detecting AGC1 or MKRN1 (red) and Mito-Tracker (green) in HCT116 cells. Scale bar: 10 μm. Western blot assay was used to detect: **(B)** the expression of AGC1 in the wild type (WT) or Oxa-resistant (OR) HCT116 cells; **(C)** AGC1 expression in HCT116 cells overexpressing vector control or Flag-AGC1 fusion protein; **(D)** Efficiency of AGC1 knockdown using lentiviral carrying shRNA.


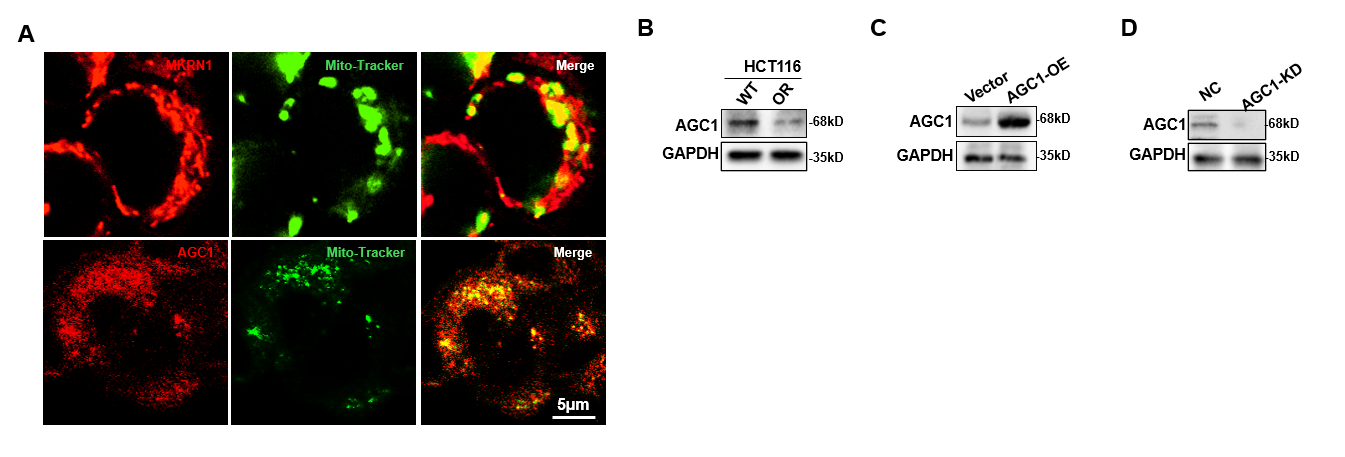

Supplement: Supplementary file 1 — Supplementary Material 1. [file 10020_2025_1287_MOESM1_ESM.docx]
